# Peripheral pulmonary hamartoma with haemoptysis from the non‐adjacent bronchus

**DOI:** 10.1002/rcr2.553

**Published:** 2020-04-06

**Authors:** Naoya Kitamura, Toshihiro Ojima

**Affiliations:** ^1^ Department of Thoracic Surgery Kurobe City Hospital Kurobe Japan

**Keywords:** Enucleation, hamartoma, haemoptysis, peripheral pulmonary thoracotomy

## Abstract

We report a rare case of peripheral pulmonary hamartoma that caused haemoptysis from the non‐adjacent bronchus. A 54‐year‐old man was admitted to our hospital because of haemoptysis. Contrast‐enhanced chest computed tomography revealed a tumour measuring 25 × 24 mm in the left lower lobe. Fluid was observed around the pulmonary artery in addition to the consolidation of the lingular segment of the left lung. Bronchoscopy confirmed continuous bleeding from the lingular bronchus, and surgery was planned. The patient was promptly diagnosed with pulmonary hamartoma intraoperatively, and tumour enucleation was performed. The haemoptysis resolved after surgery, and the patient was discharged on post‐operative day 10. As peripheral hamartoma does not generally cause haemoptysis, we suspected that the haemoptysis occurred due to the unusual condition in which the bleeding from the tumour reached the non‐adjacent lingular branch along the pulmonary artery sheath.

## Graphical abstract

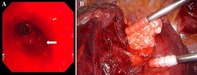

## Introduction

Pulmonary hamartoma is the most common benign lung tumour. It is usually asymptomatic and is often found incidentally. However, endobronchial hamartoma can occasionally cause haemoptysis. On the other hand, extrabronchial pulmonary hamartoma (peripheral pulmonary hamartoma) rarely causes haemoptysis, and haemorrhage from the bronchus non‐adjacent to the tumour is even rarer. Here, we report a case involving a middle‐aged man who developed a peripheral pulmonary hamartoma with haemoptysis from the non‐adjacent bronchus and exhibited resolution of the haemoptysis after tumour enucleation.

## Case Report

**Figure 1:**
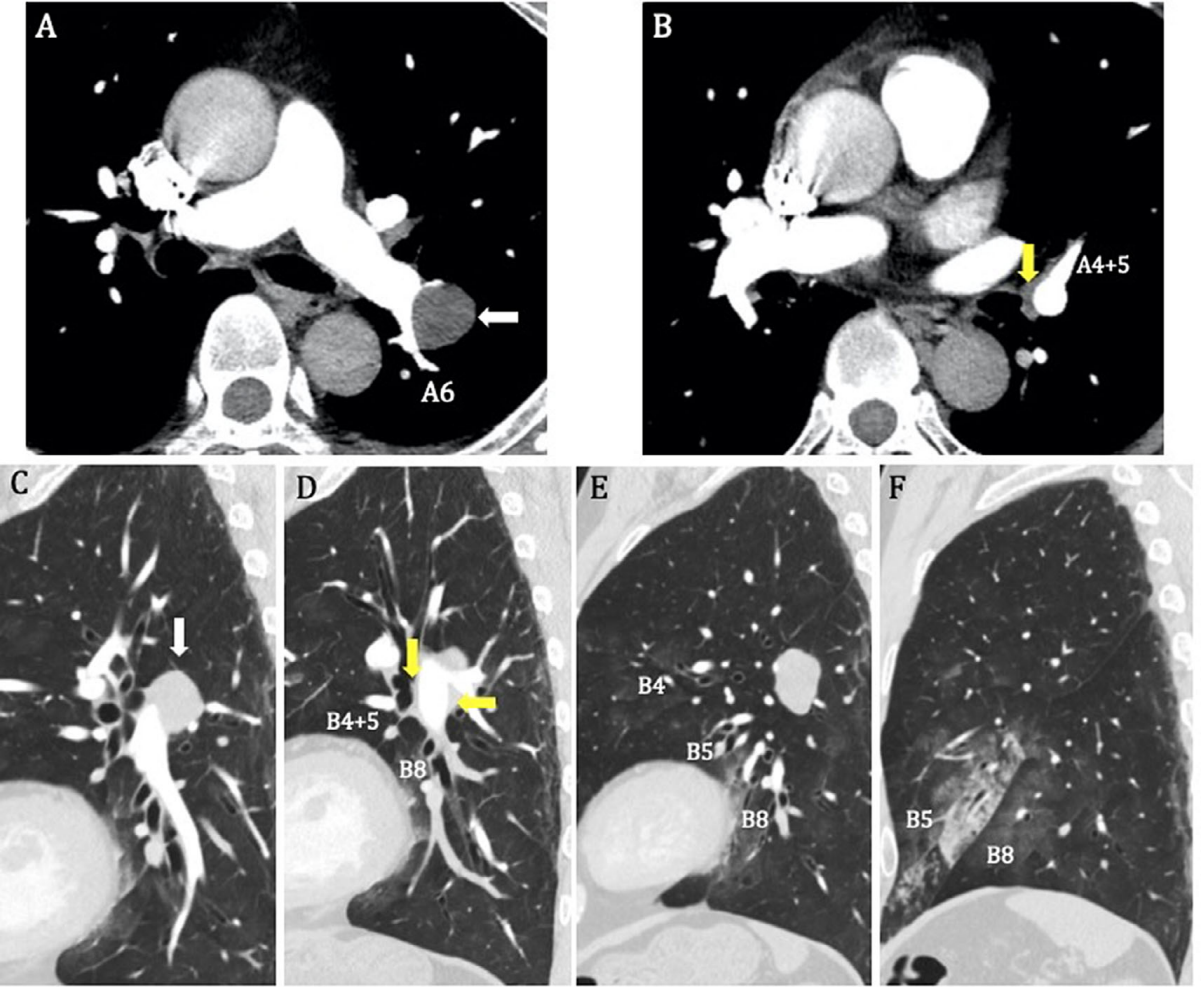
(A–D) Enhanced chest computed tomography (CT) shows a tumour measuring 25 × 24 mm in the left lower lobe (white arrow) with fluid accumulation, which suggests bleeding around the main trunk of the pulmonary artery (yellow arrow). (E, F) Enhanced chest CT showing a consolidation mainly in the lingular segment of the left lung.

**Figure 2:**
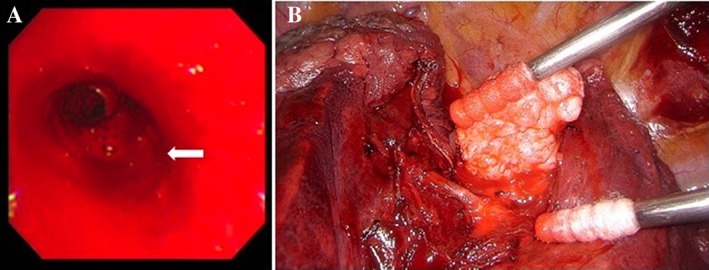
(A) Bronchoscopy showing persistent bleeding from the left lingular bronchus (white arrow). (B) Intraoperative findings showing a tumour between the upper and lower lobes, and the tumour was enucleated without obvious infiltration into the pulmonary artery.

A 54‐year‐old man was referred to our hospital with a chief complaint of haemoptysis. Chest X‐ray showed consolidation in the left lower lung field. Contrast‐enhanced chest computed tomography (CT) revealed a tumour measuring 25 × 24 mm in the left lower lobe (Fig. 1A, C). Fluid collection, which was suggestive of bleeding, was observed around the main trunk of the pulmonary artery and the lingular bronchus, not adjacent to the tumour (Fig. 1B, D). Consolidation was also observed mainly in the lingular segment of the left lung (Fig. 1E, F). Blood tests showed no increase in the inflammatory response and tumour markers. The sputum culture test was also negative, and there were no findings suggestive of infection or malignancy. The patient was admitted to our department for follow‐up. Bronchoscopy revealed persistent bleeding from the left lingular bronchus (Fig. 2A), and thrombin was used to stop bleeding. The inflow of the bronchial artery was unclear, and we suspected that bronchial artery embolization (BAE) was ineffective. Therefore, we decided to perform surgical resection. We accessed the surgical site using posterolateral thoracotomy in the fifth intercostal space. The tumour was identified between the upper and lower lobes and diagnosed as a pulmonary hamartoma by rapid intraoperative diagnosis using needle biopsy. The tumour could be removed from the pulmonary artery and lung parenchyma with its capsule. There were no findings of obvious infiltration into the pulmonary artery (Fig. 2B). The haemoptysis stopped after surgery, and the patient was discharged on post‐operative day 10.

## Discussion

Despite the existence of differences between reports, pulmonary hamartoma is a benign tumour that occurs in the bronchi in 3–20% of patients and at the periphery in the rest 1, 2. Symptoms such as cough, wheezing, and dyspnoea may be observed, but patients are usually asymptomatic and are often diagnosed incidentally on chest X‐ray. Although a few studies have reported the frequency of haemoptysis caused by intrabronchial hamartoma, Cosío et al. reported that 33.4% of patients had haemoptysis 3. Although Chauhan et al. reported haemoptysis in a patient with peripheral pulmonary hamartoma 1, it is generally believed that peripheral pulmonary hamartoma does not cause haemoptysis 1, 2.

The treatment of haemoptysis caused by tumours is divided into palliative treatment and radical modalities. Several studies have reported that BAE is effective as a palliative treatment. Fujita et al. reported that 96% of patients with non‐small cell lung carcinoma were successfully treated with BAE 4. BAE was judged to be ineffective and was not performed because the involvement of the bronchial artery was unknown in this patient. However, thrombin facilitates bleeding control and is considered a palliative treatment option for haemoptysis. On the other hand, radical treatment is divided into endobronchial treatment and thoracotomy, and thoracotomy is often indicated for peripheral tumours. The suitability of procedures such as enucleation, partial resection, lobectomy, and pneumonectomy depends on the location of the tumour. However, local resection is recommended for pulmonary hamartoma because it is a benign tumour with a good prognosis 5. We also selected enucleation as the minimum extent of resection in this case.

Preoperative chest CT showed consolidation around the lingular segment, and bronchoscopy revealed bleeding from the lingular bronchus. The tumour was not adjacent to the lingular bronchus, and the causal relationship between the tumour and bleeding was unclear. However, chest CT revealed fluid accumulation, which was thought to be caused by the hamartoma that extended from around A6 to the lingular bronchus, and haemoptysis stopped after tumour enucleation. On the basis of the results mentioned above, this case exhibited an unusual condition in which the haemorrhage from the pulmonary hamartoma reached the non‐adjacent lingular bronchus along the pulmonary artery sheath, resulting in haemoptysis. To the best of our knowledge, this is the first report of a peripheral pulmonary hamartoma with haemoptysis from the non‐adjacent bronchus that resolved after enucleation of the tumour.

### Disclosure Statement

Appropriate written informed consent was obtained for publication of this case report and accompanying images.
